# Seminal Plasma Activity to Improve Implantation in *In Vitro* Fertilization—How Can It Be Used in Daily Practice?

**DOI:** 10.3389/fendo.2018.00208

**Published:** 2018-04-27

**Authors:** Frank Nawroth, Michael von Wolff

**Affiliations:** ^1^Centre for Infertility, Prenatal Medicine, Endocrinology and Osteology, Amedes, Hamburg, Germany; ^2^Division of Gynecological Endocrinology and Reproductive Medicine, University Women’s Hospital, Bern, Switzerland

**Keywords:** seminal plasma, implantation, *in vitro* fertilization, sperm preparation, intravaginal, intracervical

We have recently published a meta-analysis of 8 randomized controlled trials (RCTs) including 2,128 women and described that intravaginal or intracervical application of ejaculate and seminal plasma (SP) around the time of oocyte aspiration and/or embryo transfer in IVF therapies significantly increased clinical pregnancy rate (RR 1.20, 95% CI 1.04–1.39) (1). These results confirmed and extended an earlier meta-analysis which had also shown significantly improved outcomes when women are exposed to SP around the time of ovum pick-up or embryo transfer with statistical significance for clinical pregnancy rate (2). In our meta-analysis, we had added a subgroup analysis with 4 RCTs including 780 participants in which prepared undiluted SP was injected intravaginally/intracervically just after oocyte aspiration. This analysis revealed an increased pregnancy rate in women treated with SP (RR 1.23, 95% CI 1.05–1.45) (1), an effect that seems to be independent from the quality of sperm count (3). By contrast, intrauterine application of diluted SP did not show any positive effect (4).

Therefore, local application of undiluted SP or its constituents can now be considered as a straightforward, non-invasive, and rather cheap treatment to improve implantation in IVF. The rationale of this treatment is the modulation of endometrial function (5), which has been shown to be negatively affected in IVF (6). Furthermore, SP might positively modulate the maternal immune system to better accept the semi-allogenic embryo (7).

Therefore, this approach could be considered to be integrated in daily practice in IVF with fresh embryo transfer. However, this raises the question of which approach would most reasonably be expected to yield the highest effect with the lowest cost and burden for the couple, the IVF laboratory, and the physician.

Four options could be considered:

1. Defined constituents of SP such as TGFβ are used instead of prepared SP.
2. The couple is instructed to have intercourse around the time of follicle aspiration and/or embryo transfer.
3. Undiluted SP is injected into the vagina/cervix just after follicle aspiration.
4. Diluted SP is injected into the vagina/cervix just after follicle aspiration.

Ad 1: In mice it has been shown that the most relevant constituent in SP is TGFβ (8). TGFβ has immune-deviating properties which favor implantation (9). In humans, TGFβ is also a major constituent of SP which influences cervical immune function *in vitro* (10). Accordingly, using TGFβ instead of SP appears to be a straightforward option as TGFβ seems to play a role in the modulation of the immune response and thereby possibly improve implantation. However, TGFβ has only been proven in mice to play a role in implantation but convincing data in humans regarding implantation are still missing. Furthermore, Gutsche et al. (5) have shown in humans that although TGFβ seems to play an important role it is rather the combination of several constituents which provokes the implantation improving effect. This is in line with Sharkey et al. (10) who demonstrated that although TGFβ is able to induce the production of IL-6 and GM-CSF in cervical cells the induction of several other cytokines is not related to TGFβ. Therefore, substituting SP by specific SP constituents might be an option but could result in lower efficacy.

Ad 2: This option seems to be the easiest one. However, the abovementioned subgroup analysis studying the application of undiluted SP into the vagina/cervix revealed a significantly improved pregnancy rate, whereas an RCT analyzing the effect of intercourse did not find a significant difference (11). Tremellen et al. (11) did find a higher proportion of viable embryos, but the pregnancy rate was not higher in the intercourse group. This suggests that intercourse might increase the likelihood of early embryo implantation but that vaginal/cervical application of undiluted SP is more effective. Furthermore, as intercourse after ovarian hyperstimulation can be expected to be painful and as there is a risk of multiples if not all follicles were aspirated, the application of SP seems to be more effective and safer than intercourse.

Ad 3: This option involves the collection and preparation of SP a few days or weeks before aspiration followed by cryopreservation, as described by the first pilot by von Wolff et al. (12), requiring an extra consultation of the male and an extra effort and additional costs for the lab. For this approach, semen samples should be obtained from the patient’s partner by masturbation, at least a half-week before follicle aspiration, and collected into sterile flasks. SP is then extracted by centrifugation and cryopreserved until instilled into the female tract at the time of follicle aspiration or embryo transfer (12). As the approach had been chosen by the other clinical studies included in the abovementioned subgroup analysis, it can be expected to be effective.

Ad 4: Option 3 also raises the question as to whether it is perhaps possible to avoid the freezing of SP a few days or weeks before aspiration. For example, small amounts of fresh SP, isolated from the ejaculate used for the IVF/ICSI procedure on the day of follicle aspiration, could be collected before density centrifugation to prepare it for intravaginal/intracervical application. This approach may be achievable in men with sufficiently high sperm concentrations and ejaculate volume; however, in men with low sperm concentrations or low ejaculate volume, this would not be possible. This option would avoid an extra consultation with the male partner and thereby better integrate this procedure into daily IVF practice. However, in men with a low sperm count and/or semen volume, the whole ejaculate volume would be needed for sperm preparation, and the SP collected after density centrifugation will contain a substantial amount of preparation medium with unknown effects on the activity of SP and the female genital tract. Furthermore, this option would increase the workload for the lab at a time when the lab is already maximally involved with follicle aspiration and oocyte preparation. There are more points which question this approach. First, Gutsche et al. (5) found that the stimulatory effect of SP decreased with lower SP concentrations, questioning the effect of low SP concentrations. Clinically, this was confirmed by von Wolff et al. (4), who did not find any positive effect with intrauterine instillation of 1:5 diluted SP. Furthermore, the concentration of active constituents such as cytokines varies considerably in SP as shown by Huleihel et al. (13) and others. Therefore, the implantation improving effect might be even lower in some subjects with low concentration of active constituents. Therefore, this approach does not seem to be feasible.

In conclusion, the application of undiluted SP seems to be more effective and safer than intercourse at the time of follicle aspiration. SP should, to be used in daily practice, be isolated and prepared at least a few days before oocyte aspiration and used without any dilution. Determination of the most beneficial time for instillation of undiluted SP during an IVF/ICSI cycle is another important parameter to be considered in future RCTs. More research is required before modifications of this approach such as the use of specific SP constituents or of diluted SP can be recommended. Further RCTs should be encouraged to confirm the positive effect of diluted/undiluted SP and to better understand under what circumstances this treatment has the best clinical outcome.

## Author Contributions

Both authors analyzed the data and wrote the paper.

## Conflict of Interest Statement

The authors declare that the research was conducted in the absence of any commercial or financial relationships that could be construed as a potential conflict of interest.
